# Synchrony During Online Encounters Affects Social Affiliation and Theory of Mind but Not Empathy

**DOI:** 10.3389/fpsyg.2022.886639

**Published:** 2022-07-11

**Authors:** Chiara Basile, Serena Lecce, Floris Tijmen van Vugt

**Affiliations:** ^1^Department of Brain and Behavioral Sciences, University of Pavia, Pavia, Italy; ^2^Department of Psychology, University of Montreal, Montreal, QC, Canada; ^3^International Laboratory for Brain, Music and Sound Research BRAMS, Montreal, QC, Canada; ^4^Centre for Research on Brain, Language and Music – CRBLM, Montreal, QC, Canada; ^5^Haskins Laboratories, Yale University, New Haven, CI, United States

**Keywords:** synchrony, theory of mind, empathy, social affiliation, online meetings, closeness

## Abstract

Moving together in time affects human social affiliation and cognition. However, it is unclear whether these effects hold for on-line video meetings and whether they extend to empathy (understanding or sharing others' emotions) and theory of mind (ToM; attribution of mental states to others). 126 young adult participants met through online video in unacquainted pairs. Participants either performed 3 min of synchronous arm movements paced by sounds (*n* = 40), asynchronous movements (*n* = 46) or a small talk condition (*n* = 40). In a subsequent empathy task, participants engaged in a conversation. A video recording of this conversation was played back, and each participant rated, at predetermined time points, how they felt and how they thought their partner felt. From this we calculated empathic accuracy (accuracy of the estimation of the other's emotions) and emotional congruence (emotion sharing). ToM was measured by showing videos of geometrical shapes interacting and asking the participants to describe what happened, measuring the amount of intentionality. We found that participants in the synchrony condition rated feeling greater closeness and similarity to their partners relative to the asynchronous condition. Further, participants in the synchrony group tended to ascribe more intentionality to the abstract shapes than participants in asynchrony condition, suggesting greater ToM. Synchrony and asynchrony groups did not reliably differ in empathic accuracy nor emotional congruence. These results suggest that moving in synchrony has effects on social affiliation measures even in online encounters. These effects extend to ToM tendencies but not empathic accuracy or emotion sharing. These results highlight the potential of synchronous movement in online encounters to affect a subset of social cognition and affiliation measures.

## Introduction

People moving in synchrony occurs in cultures across the globe in activities ranging from rituals and ceremonies to military marching (McNeill, 1997). Synchronous movement appears early in life (Cirelli, 2018). Synchronization can occur spontaneously during an interaction or can be intentional as when ensemble musicians adjust their movements to each other (bidirectionally) or when orchestra members follow the beat imposed (unilaterally) by a conductor. Synchrony has a wide range of effects on human social cognition, promoting social bonding (Huron, 2001) and enhancing social cohesion (Hove and Risen, 2009; Wiltermuth and Heath, 2009) and cooperation (Wiltermuth and Heath, 2009) both within and outside behaviorally synchronous groups (Reddish et al., 2014; Sullivan et al., 2015) (but see also Gelfand et al., 2020). The aim of the present study was to investigate whether these effects hold for online encounters between unacquainted pairs of young adults and whether these effects extend to more complex socio-cognitive abilities such as theory of mind (e.g., ToM) and empathy.

Moving in synchrony with somebody, whether spontaneous or imposed, has previously been shown to increase the sense of connection and liking (Hove and Risen, 2009; Tarr et al., 2015, 2016) and boost individual perception of closeness and feelings of similarity (Paladino et al., 2010; Valdesolo et al., 2010; Vacharkulksemsuk and Fredrickson, 2012; Reddish et al., 2013; Tarr et al., 2016). Synchrony has also been shown to increase positive affect (Tschacher et al., 2014) and to improve memory recall of words spoken by the person that one synchronizes with (Macrae et al., 2008). Drawing on a meta-analysis including more than 4,000 participants, Mogan and colleagues concluded that moving in synchrony increases four main dimensions of social relationships: social behaviors, such as prosocial actions, social bonding, including the feeling of similarity and closeness, social cognition, such as the ability to memorize or paying attention to others, and positive affect, such as mood, happiness, self-esteem, and general life satisfaction (Mogan et al., 2017). Altogether these studies show that synchrony affects several dimensions of social affiliation such as closeness, prosocial behavior, and perceived similarity. Studies in this field have been typically conducted in person with participants being in the same room while performing the synchronous movements. Therefore, it is not clear whether synchrony can also affect social affiliation when participants meet online rather than in physical presence. This is not a trivial question as there are a number of key differences between online (video) and in-person meetings which may influence the social dynamics of these encounters (Day and Schneider, 2002). First of all, the fixed camera in online meetings forces people to stay relatively still in order to be visible hindering the regulation of social distance which is known to be an important part of natural conversation (Patterson, 1996). Second, in online encounters some sensory information is not shared between the interacting partners such as external sounds and odors, which can have an impact on perceived interaction and emotional engagement with the other person (Johnson et al., 2006; Sohn, 2011). These features may explain why it is more difficult to recreate a sense of 'being there together' in online encounters (Parkinson and Lea, 2011). Given the differences between online and in-person social interactions, we cannot assume that the effects of synchrony on social affiliation, which have been tested virtually exclusively in-person, generalize to online interactions. The first aim of the present study is to test whether, in online video meetings, synchrony affects social affiliation.

The second set of questions we address relates to socio-cognitive skills. Recently it was suggested that the effects of synchrony extend beyond social affiliation (e.g., closeness, social bonding) to also include people's socio-cognitive skills (Baimel, 2015). In particular, the present study focuses on the tendency to attribute mental states and intentionality to others, typically referred to as ToM, on the one hand, and on the accuracy of perceiving other's emotions and on the extent of sharing them, typically referred to as cognitive and affective empathy, respectively.

Connections between synchrony and empathy have been investigated, focusing predominantly on subjective reports. In one study participants rhythmically moved cups in time with sounds presented through headphones (Baimel et al., 2018). They did so in trios where either the sounds were synchronous (i.e., same tempo for all participants) or asynchronous (i.e., different tempi for different participants). As a result, participants performed movements at the same time in the synchrony but not in the asynchrony condition. Participants moving synchronously rated themselves as better in understanding others' emotions, but not in sharing these emotions, relative to participants moving asynchronously (Baimel et al., 2018). This latter result differs from Koehne et al. (2016) who tested participants in a unilateral synchrony task where participants, in a leader role, were followed by a computer algorithm with a high or low degree of synchrony. Participants (healthy controls) who were followed synchronously self-reported higher affective empathy (i.e., sharing emotions) relative to those who were followed asynchronously. Taken together, synchrony increases subjective understanding of the emotions of others (cognitive empathy) and possibly also self-reported sharing of the emotions of others (affective empathy). However, these studies relied on self-reported empathy measures. There are often considerable discrepancies between self-reported measures of empathy (e.g., using questionnaires) and objective measures of people' ability to read and share emotions. Several studies found little to no correlation between objective and self-reported empathy measures (Levenson and Ruef, 1992; Ickes, 1993; Realo et al., 2003; Jospe et al., 2020). Indeed, only a small portion of the variance in objective measures may be explained by self-reported questionnaires: around 1% according to some estimates (Murphy and Lilienfeld, 2019). This is not surprising if we consider that self-report questionnaires (but not objective measures) rely on metacognitive insight into one's capacities and are sensible to well-documented biases: social desirability (Sedikides et al., 2003) and the Dunning-Kruger effect (where poor performers overestimate their abilities and high performers often underestimate their abilities, Ames and Kammrath, 2004).

The effect of synchrony on objective measures of empathy remains less clear. The discrepancy between objective and self-reported empathy measures implies that we cannot simply extrapolate the findings from self-report measures to objective empathic performance. Studies that did use objective measures of empathy tended not to find effects of synchrony. For example, synchronous movements did not significantly affect participants' ability to attribute the correct emotions to a corresponding image of the eye region (Baimel et al., 2018). Using this same Reading the Mind in the Eyes task, Koehne et al. (2016) found no correlation with perceived synchrony (in a unilateral synchrony task). These findings are important but leave open one possibility. The studies had participants rate emotions of unknown people in pictures, whereas the effects of synchrony may be specific to the synchronizing partners. Indeed, the issue of whether the effects of synchronous movements are specific to those we synchronize with is still open: some studies report that synchrony selectively boosts helping behaviors toward the people we synchronize with (Cirelli, 2018) while others show that moving in synchrony induces an effect that generalizes even to those who did not move in synchrony with us (Reddish et al., 2014, 2016). Thus, in the case of empathy, one would need to measure the accuracy or reading the emotions of the people that one synchronizes with. This is exactly what was done more recently, in a study using the Empathic Accuracy paradigm in which two partners first engage in a conversation and are then asked a series of questions on their own and their partner's feelings and thoughts. The study found that the degree of success in inferring others' emotions and thoughts (empathic accuracy) was not significantly associated with spontaneous behavioral synchrony during the conversation (Fujiwara and Daibo, 2021). Although highly relevant, this study relied on spontaneous behavioral synchrony which tends to be less stable than imposed synchrony (Richardson et al., 2005, 2007; Schmidt and Richardson, 2008). For this reason, it is possible that the resulting effects of synchrony on empathy were too small to detect. Imposing synchrony in a stable manner may affect empathic accuracy more robustly, as we will test here. Further, the Fujiwara and Daibo study measured accuracy for inferring both emotions and thoughts and so the absence of effect may be because synchrony has a different effect on awareness of thoughts (Theory of Mind) and awareness of emotions (empathy).

Studies on empathy have focused primarily on cognitive (emotion understanding) rather than affective (emotion sharing) empathy. Theoretical accounts predict that behavioral synchrony should lead to alignment of affective states (Shamay-Tsoory et al., 2019) but to our knowledge, this has been tested using subjective, not objective measures of emotion sharing. Using subjective measures, Koehne et al. (2016) found that participants self-reported more emotion sharing with synchronous vs. asynchronous partners. However, again this is a self-report measure, and it remains unclear whether when measured objectively, synchronous participants share more emotions.

In sum, synchrony affects subjective cognitive empathy, but no effects have been found using objective measures, possibly because they used spontaneous synchrony or stimuli other than the person we synchronize with. In the present study we asked dyads to synchronize with a metronome and then measured empathy toward the synchronization partner in order to investigate whether objective empathy (both cognitive and affective) is influenced by synchrony.

The present study also addresses the question of whether moving in synchrony affects tendency to attribute mental states to others, namely Theory of Mind (ToM) (Wimmer and Perner, 1983). In this study we explore this issue in adult participants whereas prior work on ToM, independent of synchrony, focused predominantly on children and atypical populations. Recent work shows that ToM continues to develop during adulthood (Apperly et al., 2010; Klindt et al., 2017) and new tasks have been developed to assess adults' actual and self-reported advanced ToM skills (Apperly, 2011; Devine and Lecce, 2021). Previous research offers some preliminary evidence that synchrony affects self-reported mentalizing. For example, participants who in small groups moved synchronously to a metronome increased the extent to which mental states are ascribed to the other members of the group (Baimel et al., 2018). Similarly, perceived synchrony, in a unilateral synchronization experimental task, correlated with the perceived ability to understand the thoughts and intentions of their partner (Koehne et al., 2016). Together, these studies suggest that moving in synchrony with a partner increases self-reported understanding of the partner's mental states. It remains unclear whether synchrony affects people's actual attribution of mental states using performance-based measures of ToM. Performance-based tasks differ from self-report questionnaires in that they do not assess people's beliefs about their own ToM but, rather, examine the extent to which people actually attribute mental states to selected stimuli such as characters in vignettes (e.g., Happé, 1994) or videos (e.g., Murray et al., 2017), or to abstract geometrical moving shapes (e.g., White et al., 2011). While emerging research has shown that self-reported questionnaires may correlate with performance-based measures (Bukowski and Samson, 2017; Clutterbuck et al., 2021), it is not clear whether the effects of synchrony found in self-reports extend to performance-based ToM measures. To date the only study conducted on this topic is that of Koehne et al. (2016). After having performed the unilateral synchrony task (see above), participants completed the Movie to Assess Social Cognition (MASC), a video-based task in which participants watch a short movie about four characters getting together for a dinner party and to answer to a series of questions concerning the characters' mental states (Dziobek et al., 2006). Authors found that individual performance in produced synchrony (follower's success in adjusting his or her movements to the leader to produce synchrony) were unrelated to performance in the MASC. Two features of this study should, however, be noted here. First, correlations are based on a restricted sample of 22 healthy controls; second, as the authors pointed out, there may have been too low variability in MASC scores to detect correlations with synchrony. In the present study we aimed to recruit a larger sample and employ a different performance-based ToM task to test whether synchrony affects ToM. The task chosen is the Triangle task (Castelli et al., 2000), that measures participants' attribution of intentionality as they describe a video of moving geometric shapes in absence of any contextual verbal or non-verbal cues other than movement.

In the present study we recruited participants who were divided into dyads that were unacquainted. These dyads met online through video conference and performed a series of periodic hands movement (clapping) paced by a metronome, either in synchrony or asynchrony with their partner. However, both these conditions involved rhythmic arm movements and fixed gaze position that may be perceived as unnatural. As a result, participants could experience these conditions as awkward on the one hand or as fun and engaging on the other hand. Such perceptions could affect the social affiliation measures that we collect here. Thus, to put the results from the synchrony and asynchrony conditions in perspective, we included a more conventional interaction: the small talk condition in which participants freely talked about a set of predefined questions. In this group, participants were not restricted in their arm, head and eye movements. Thus, this condition serves as a relatively ecological baseline to which any differences between synchrony and asynchrony can be interpreted. The aims of the study were to examine the effect of synchrony on (1) social affiliation (perceived similarity, closeness, likeability, and future friendship), (2) the tendency to attribute mental states and intentionality to others (ToM), and (3) on the accuracy of perceiving other's emotions and sharing them. Empathy was measured using the empathic accuracy paradigm, an ecological task that constitutes a relatively objectively measure of whether participants in dyads can accurately infer each other's emotions (cognitive empathy) and share those emotions (affective empathy). ToM was measured using two tasks: a self-report questionnaire (measuring the extent to which mental states were attributed to the dyadic partner) and a performance-based measure (measuring the degree of intentionality ascribed to abstract moving shapes in a video). The study was conducted entirely online during the COVID-19 pandemic in Italy (spring 2020) which was a period of a degree of imposed social isolation. To control for potential effects of this social isolation, we measured social contacts, wellbeing, and loneliness.

## Materials and Methods

### Participants

One hundred twenty-six Young Adults Were Recruited Through Social Networks and the Newsletter of the Psychology Department. Participants Had a Mean age of 23.59 years (SD = 3.21, Range = 19–32 years) and Were Randomly Assigned to one of the three Experimental Conditions: Synchrony (*N* = 40; 30 Females), Asynchrony (*N* = 46; 35 Females) and Small Talk (*N* = 40; 30 Females). All Participants Were Fluent Italian Speakers. Criteria for Inclusion Were Written Consent and age Between 19 and 35 years. The Study Was Approved by the Ethics Committee of the Department of Brain and Behavioral Sciences of the University of Pavia (Approval # 048/20). Prior to Participation, all Participants Were Informed About the Aims of the Study and Signed the Informed Consent, According to the Declaration of Helsinki. The Dataset Is Available Online at osf.io/Jchzb/.

### Procedure

**Figure 1:**
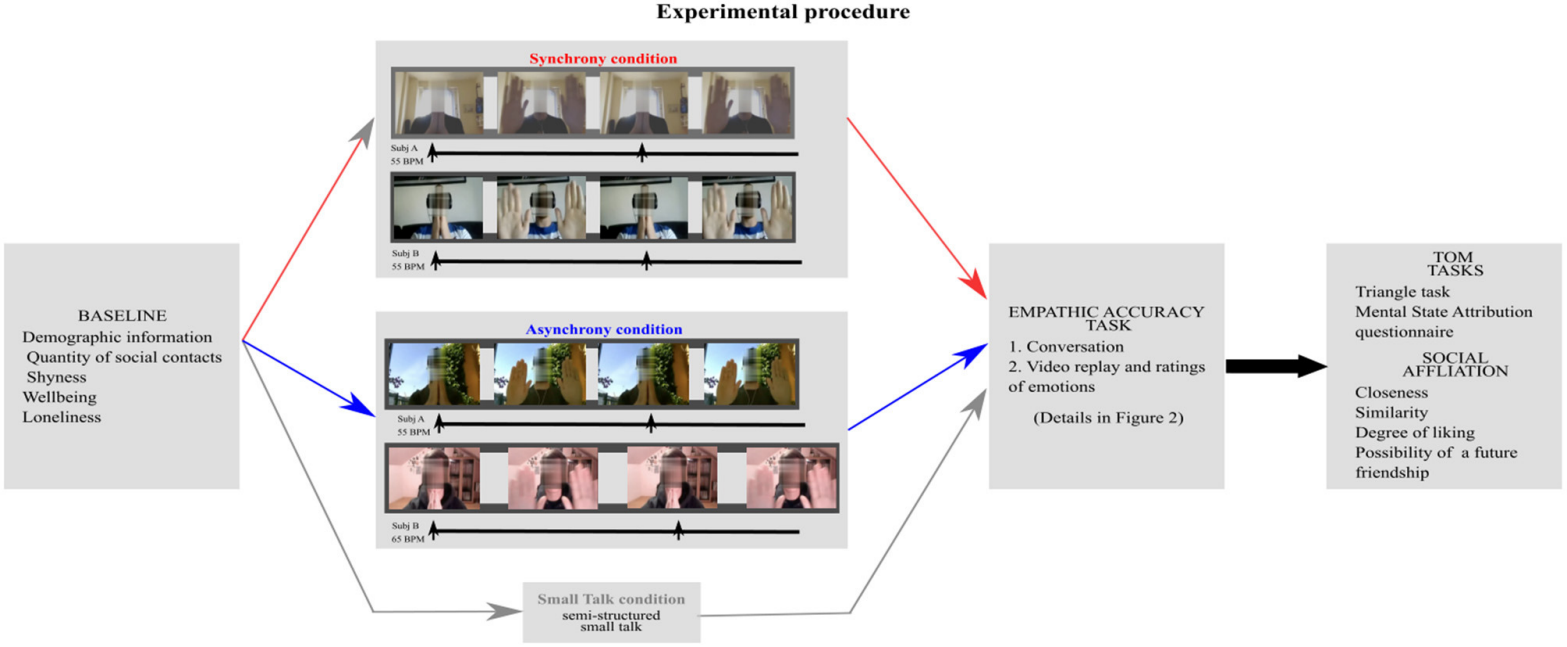
Experimental procedure. Participants first completed a questionnaire assessing baseline variables, second, engaged in synchronous/asynchronous movement or small talk, third, completed the Empathic accuracy procedure and finally, completed the ToM tasks and a series of questions evaluating social affiliation. In the synchrony and asynchrony conditions subjects clapped their hands in time with a metronome sound presented *via* headphones while looking at their partner. In the synchrony condition the metronome had the same BPM (beats per minutes) for both partners. In the asynchrony condition the metronome had a different BPM for the partners. Movements were therefore aligned in time in the synchrony but not in the asynchrony condition. In the figure the lines represent the time and the arrows represent when the metronome sound occurred, in time with which participants clapped their hands. In the small talk condition, participants took part in a semi-structured conversation.

Participants were contacted *via* mail and asked to complete a questionnaire assessing baseline variables: demographic information, quantity of social contacts, shyness, wellbeing, and loneliness. In the second phase, participants were paired in dyads according to the following rules: being unacquainted, close in age (maximum age difference was 3 years) and of the same gender. Dyads were randomly attributed to one of three experimental conditions: synchrony, asynchrony, and small talk. After the manipulation, all participants, first, took part in the empathic accuracy procedure, then completed the two ToM tasks (the Triangle task and the Mental State Attribution questionnaire) and, finally, filled out a series of questions evaluating social affiliation (closeness, similarity, degree of liking, and possibility of a future friendship; details below). The experimental procedure is represented in Figure 1.

The entire video call took ~90 min.

#### Synchrony and Asynchrony Manipulation

In the synchrony condition participants were asked to make a cyclic movement with their hands (touching the palms of the hands and then putting them opened in front of the camera) in time with a metronome (periodic beep) sound that was presented through Zoom at the same tempo for both partners (65 beats per minute (bpm) for half the dyads and 55 bpm for the other half). These particular tempi were chosen for consistency with prior studies (Reddish et al., 2013, 2014). Participants were instructed to perform the movement continuously while looking at their partner. In the asynchrony condition participants received the same instructions and followed the same procedure as those belonging to the synchrony group. The only difference was that in the asynchrony condition, the two participants were received a different tempo (55 bpm and 65 bpm) (see Figure 1) and as a result their movements did not generally align in time. Participants in both these conditions were not allowed to talk to each other.

Immediately following the movements, participants were asked whether they had perceived Internet connection problems. Results showed that 13 participants in the synchrony group (32.5%) and 14 in asynchrony group (30.4%) reported that they perceived Internet connectivity issues. These numbers were not significantly different between groups (*X*^2^(2,85) = 0.08, *p* = 0.78).

#### Small Talk Condition

In the small talk condition participants (instead of performing movements either in synchrony or asynchrony with their partner) took part in a semi-structured conversation based on 24 questions of the small talk task developed by Sedikides et al. (1999). The subjects were instructed to go through the 24 questions in order, taking turns answering one question. Participants were not restricted in their movements, and they could see each other during the conversation.

#### Empathic Accuracy

**Figure 2:**
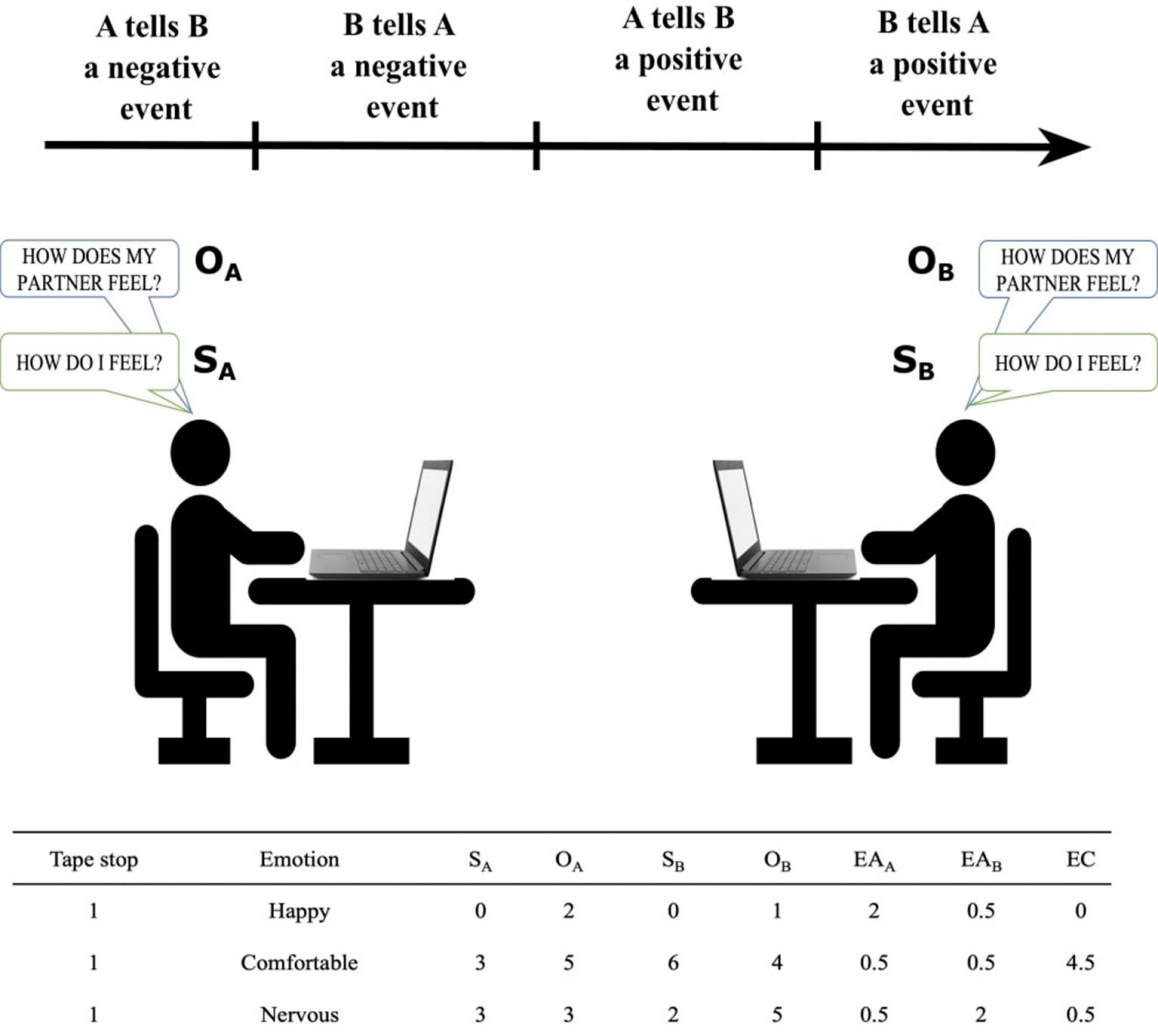
Empathic accuracy procedure and an example of empathic scores computation. Two participants (A, B) took part in a semi-structured conversation in which both participants talk about positive and negative events in their life. At the end of talk, the researcher showed participants the recorded video of their conversation twice, interrupting the video at eight time points (tape stops). At each tape stop, the researcher asked participants to rate their own (in the first viewing) and their partner's (in the second viewing) emotions. Based on these answers, we computed empathic accuracy (EA) and emotional congruence (EC) as shown, for an example portion of the data in the table. S_A_, self-reported feelings of participant A; S_B_, self-reported feelings of participant B; O_A_, other-rating of A: estimation by A of B's feelings; O_B_, other-rating of B: estimation by B of A's feelings; EA_A_, empathic accuracy of participant A; EA_B_, empathic accuracy of participant B; EC, emotional congruence of the dyad.

The empathic accuracy procedure (adapted from Blanke et al., 2016) consists of a semi-structured conversation in which participants are asked to talk, in turn, for 3 min about a positive and a negative event that happened in their life (Figure 2). The listener is allowed to interrupt the partner to ask questions. At the end of this 12-min conversation, the researcher showed participants the recorded video of their conversation twice, interrupting the video every 90 s (“tape stop”). At each tape stop, the researcher asked participants to report their own (in the first viewing) and estimate their partner's (in the second viewing) emotions answering the following questions: “How do you feel?” and “How does your partner feel?”. Participants answered these questions for each of nine emotions (five positive: happy, excited, content, comfortable, balanced and four negatives: nervous, sad, uncomfortable, tense) using a 7-point scale ranging from 0 (not at all) to 6 (very much) (see Measures). The answers given by the subjects were confidential (invisible to their dyadic partner) (see Figure 2). After completing the empathic accuracy procedure, participants were administered the two ToM tasks, the Triangle task and the Mental State Attribution questionnaire, and the social affiliation questionnaires.

### Measures

#### Baseline Variables

##### General Information Questionnaire

We asked participants to report on their age, gender, and general information about the social situation that they were experiencing (i.e., days of isolation, number of times they went out during the last week).

##### Social Contacts

We administered an in-house questionnaire to assess the frequency of social contacts (e.g., phone call, texting, and videocalls) during the last week. Possible answers range from 0 (never) to 4 (more than once a day) and were summed into a total score ranging from 0 to 70 (α = 0.64).

##### Wellbeing

We used the Italian version of the Warnick-Edinburgh Mental Well-Being Scale (WEMWBS; Gremigni and Stewart-Brown, 2011). WEMWBS is a 14-item scale covering both hedonic and eudaimonic aspects of mental health including positive affect (feelings of optimism, cheerfulness, and relaxation), satisfying interpersonal relationships and positive functioning (energy, clear thinking, self-acceptance, personal development, competence, and autonomy). Participants were required to tick the box which best described their own experience over the past 2 weeks using a 5-point Likert scale (1 = none of the time; 5 = all of the time). Item scores were summed into a single total score of wellbeing ranging from a minimum of 14 to a maximum of 70, with higher scores representing higher levels of mental wellbeing (α = 0.85).

##### Loneliness

Participants filled out the Revised UCLA Loneliness Scale (R-UCLA; Russell et al., 1980). This scale consisted of 20 items, 10 positively (e.g., “There are people I can turn to”) and 10 negatively worded (e.g., “I feel isolated from others”). Answers were based on a 4-points Likert scale ranging from 1 (never) to 4 (often). Once items were reverse scored, all of them were summed to create an overall index of loneliness, ranging from a minimum of 20 to a maximum of 80 with higher scores indicating greater loneliness (α = 0.79).

##### Shyness

Participants responded to the Italian version of the Revised Cheek and Buss Shyness Scale (RCBS-14; Marcone and Nigro, 2001). The scale consists of 14 items to which subjects responded on a 5-point Likert scale from 0 (completely disagree) to 4 (completely agree). Responses were summed into a single shyness score (range 0–56) (α = 0.89).

#### Variables of Interest (Post-manipulation)

##### Empathy

This was measured using the empathic accuracy task (adapted from Blanke et al., 2016) which consists of a semi-structured conversation between two participants (see procedure 2.2.1). On the basis of participants' answers to the questions of “How do you feel?” and “How does your partner feel?,” we computed a score of *empathic accuracy* as the similarity between the participant's self-reported feelings and the empathizer's judgment of the experimental partner's feelings (cognitive empathy) and *emotional congruence* as the similarity between the two participants' self-reported feelings (affective empathy) (Figure 2). Specifically, for empathic accuracy, we calculated the sum of squared differences of the judgment of the empathizer and the self-report of the partner across the eight tape stops for each emotion, which were then averaged to yield one empathic accuracy score for positive and one for negative emotions in each participant. The reason positive and negative emotions were kept separate is because empathy for positive and negative emotions are considered as two distinct but related constructs with different properties and correlates (Rothbart et al., 2000; Sallquist et al., 2009). Analogously, emotional congruence was calculated *via* the sum of squared differences between both partners' emotion ratings across the tape stops (see Figure 2). Note that while empathic accuracy yielded an individual score for each participant, emotional congruence yielded a single score per dyad.

##### Theory of Mind (ToM)

ToM was measured using a self-report questionnaire, the Mental State Attribution questionnaire (Baimel et al., 2018), and a performance-based task, the Triangle task (Castelli et al., 2000).

The Mental State Attribution questionnaire consists of 15 items evaluating participants' tendency to view the experimental partner as someone who owns mental states (emotions, thoughts, desires) (Baimel et al., 2018). Responses were on a 7-points scale (1 = completely disagree; 7 = completely agree; summed score range: 15–105; α = 0.87).

The Triangle task (Castelli et al., 2000) evaluates the extent to which people attribute intentionality to geometric shapes on the basis of their movements. It has been shown to reliably differentiate between high-functioning ASD groups and verbal ability matched control groups (Abell et al., 2000; Murray et al., 2017) and has been used in studies with neurotypical adults (for example Devine and Hughes, 2019). From the three categories of videos (random, goal-directed and ToM) in the original study, the present study selected two ToM videos of moving geometric shapes (triangles) that behaved in such a way as to imply teasing and joking. Each animation lasted ~40 s. After watching each video clip, participants were asked to write down what happened in each clip. The score for each clip reflected the degree to which participants described the video in terms of complex intentional mental states, according to the original guidelines (Castelli et al., 2000, Appendix II, Intentionality score). The intentionality score for each description ranged from 0 (no deliberate action, e.g., “bouncing”, “rotating”) to 5 (deliberate action aimed at affecting another's mental state, e.g., “persuading”, “pretending”, and “deceiving”). Two raters independently coded 25% of the responses and interrater agreement was established using Cohen's kappa (κ = 0.77). All remaining responses were coded jointly by the raters. Disagreements were resolved through discussion between the raters. During this entire procedure the coders were blinded to the experimental condition. The summed score for the two videos could range from 0 to 10 points.

##### Closeness

We assessed how close each participant felt to their experimental partner using the Inclusion of Other in the Self Scale (IOS) (Aron et al., 1992). Participants were asked to report how close they felt to the partner by picking one out of seven “Venn diagrams”: each was a pair of more or less overlapping circles (**Figure 4D**). On one end of the continuum, the circles are completely separate, and, on the other end, the circles are virtually entirely overlapping (Range: 1–7 with higher values meaning greater closeness).

##### Perceived Similarity, Liking and Future Friendship

Participants rated on a 9-point scale from 1 (not at all similar) to 9 (very similar) (Range: 1–9) the following statement: “How similar do you feel to the participant with whom you take part in this study?” (*perceived similarity*). Participants responded on a 9-point scale from 1 (not at all) to 9 (very much) the following two statements: “How much do you like the participant with whom you take part in this study?” (*likeability*) and “In the future, to what extent do you feel that you could be friends with the participant with whom you take part in this study?” (*future friendship*) (Sedikides et al., 1999).

### Data Analysis

We first removed outliers in each dependent variable using the boxplot rule (Tukey, 1977): a data point is considered an outlier if it is more than 1.5 times the interquartile range above the upper quartile or below the lower quartile.

We performed a randomization check by running a series of ANOVAs with experimental group (synchrony, asynchrony, and small talk) as the independent variable and each baseline variable in turn as dependent variable in order to investigate whether there were differences between experimental groups at the outset (see Table 1).

**Table 1:** Descriptive statistics and group comparisons (ANOVA) on baseline variables.

**Measure**	**Synchrony** **(*****n*** **=** **40)**			**Asynchrony** **(*****n*** **=** **46)**			**Small talk** **(*****n*** **=** **40)**			**ANOVA**	
	***M***	**SD**	**Range**	***M***	**SD**	**Range**	***M***	**SD**	**Range**	***F* (df_**num**_, df_**den**_)**	***p***
Age	23.90	2.57	19–30	23.74	3.49	19–32	23.10	3.47	19–31	0.69 (2, 123)	0.499
Education	2.60	0.78	1–4	2.50	0.59	2–4	2.58	0.84	2–5	0.22 (2, 123)	0.805
Isolation	43.37	10.27	30–68	48.50	9.28	36–85	43.69	10.04	25–65	2.72 (2, 94)	0.071
Going out	1	1.28	0–4	1.39	1.20	0–4	1.30	1.22	0–4	1.15 (2, 123)	0.320
Social	15.48	4.50	4–27	14.94	4.11	5–24	15.2	4.12	3–22	0.17 (2, 123)	0.841
Shyness	38.33	4.65	30–50	39.87	4.97	31–56	38.90	6.32	19–49	0.40 (2, 123)	0.399
Wellbeing	47.98	7.51	32–62	48.28	8.51	27–69	48.68	5.99	35–62	0.09 (2, 123)	0.500
Loneliness	51.95	4.81	40–60	52.98	3.73	46–61	52.68	3.72	43–60	0.70 (2, 123)	0.915

*ANOVA, analysis of variance; Isolation, Days of isolation; Going out, number of times they went out during the last week; Social, social contacts*.

To test our main hypotheses, we performed a series of ANCOVAs with experimental condition (synchrony, asynchrony, and small talk) as the between-participants factor, variables of interest (see details below) as dependent variables and gender as covariate (to control for gender differences). For each variable of interest, we further checked whether there was a significant correlation with any of the baseline variables (general information, social contacts, shyness, loneliness, and wellbeing) and, if so, these were included as covariates. Follow-up tests were performed using planned orthogonal contrasts: first, we investigated the effect of synchrony using a synchrony vs. asynchrony contrast and, second, we contrasted synchrony and asynchrony together vs. small talk. The rationale for the planned latter contrast is that synchrony and asynchrony conditions involve precisely prescribed rhythmic movements that are potentially experienced as unnatural and rigid, in similar ways, whereas the small talk condition is a more common, ecological interaction. For reference, separate contrasts (synchrony vs. small talk and asynchrony vs. small talk) are reported in the Supplementary Materials section. Empathic accuracy and emotional congruence were analyzed using a series of ANCOVAs controlling for gender with experimental condition (synchrony, asynchrony, and small talk) as between-participants factor and the valence of emotion (positive and negative) as within-subject factor. Where significant, we then examined the effect of experimental condition on positive and negative empathic indexes separately using ANOVAs. Finally, when the ANOVA follow-up contrasts were not significant for contrasts of interest (synchrony vs. asynchrony) in the empathic measures (empathic accuracy and emotional congruence), we calculated the Bayes Factor BF_10_ (i.e., evidence for alternative vs. null hypothesis). The Bayes Factor, when smaller than 1, quantifies the amount of evidence in favor of the null hypothesis, that there is no difference between groups. Benchmark scores: BF_10_ between 1 and 1/3 are considered to be weak, between 1/3 and 1/10 are considered moderate, and >1/10 are considered strong evidence in favor of the null hypothesis (Jeffreys, 1961).

We report $\eta_{p}^{2}$ partial effect sizes (Keppel, 1991).

## Results

### Preliminary Analyses and Descriptive Statistics

*Randomization check:* ANOVAs showed no significant differences between the three groups on any of the baseline variables (all *ps* > 0.07, Table 1), indicating that there was no evidence for group differences at the outset.

Comparing values on the wellbeing scale with normative scores, we found that for the majority of our sample, wellbeing was above clinical levels (70.6%). Of the remaining 29.3%, 12.6% of participants reported a level of wellbeing indicating a probable depression and 16.7% a possible depression.

Descriptive statistics of the post-manipulation focus variables are reported in Table 2.

**Table 2:** Descriptive statistics on post-manipulation variables.

**Measure**	**Synchrony** **(*****n*** **=** **40)**			**Asynchrony** **(*****n*** **=** **46)**			**Small talk** **(*****n*** **=** **40)**		
	***M***	**SD**	**Range**	***M***	**SD**	**Range**	***M***	**SD**	**Range**
MSA	5.90	0.60	4.53–7	5.70	0.54	4.27–6.93	5.86	0.62	4.40–7
Intentionality	7.38	1.88	2–10	6.17	2.24	1–10	6.5	2.21	2–10
IOS	3.58	1.24	1–6	2.26	1.07	1–5	3.23	1.25	1–6
Similarity	6.13	1.83	1–9	5.30	1.62	2–8	5	1.81	0–8
Likeability	8.08	0.81	7–9	7.76	1.01	5–9	7.61	0.90	6–9
Future Friendship	6.91	1.40	2–9	6.85	1.15	4–9	6.13	1.77	2–9
EA	0.87	0.35	0.31–1.80	0.87	0.32	0.24–1.46	0.72	0.40	−0.04 to 1.87
EC	0.59	0.32	0.06–1.32	0.71	0.49	−0.13 to 1.55	0.55	0.40	−0.39 to 1.25

*MSA, mental state attribution; Intentionality, theory of mind score in the triangle task; IOS, inclusion of other in self; EA, empathic accuracy; EC, emotional congruence*.

### Manipulation Check

**Figure 3:**
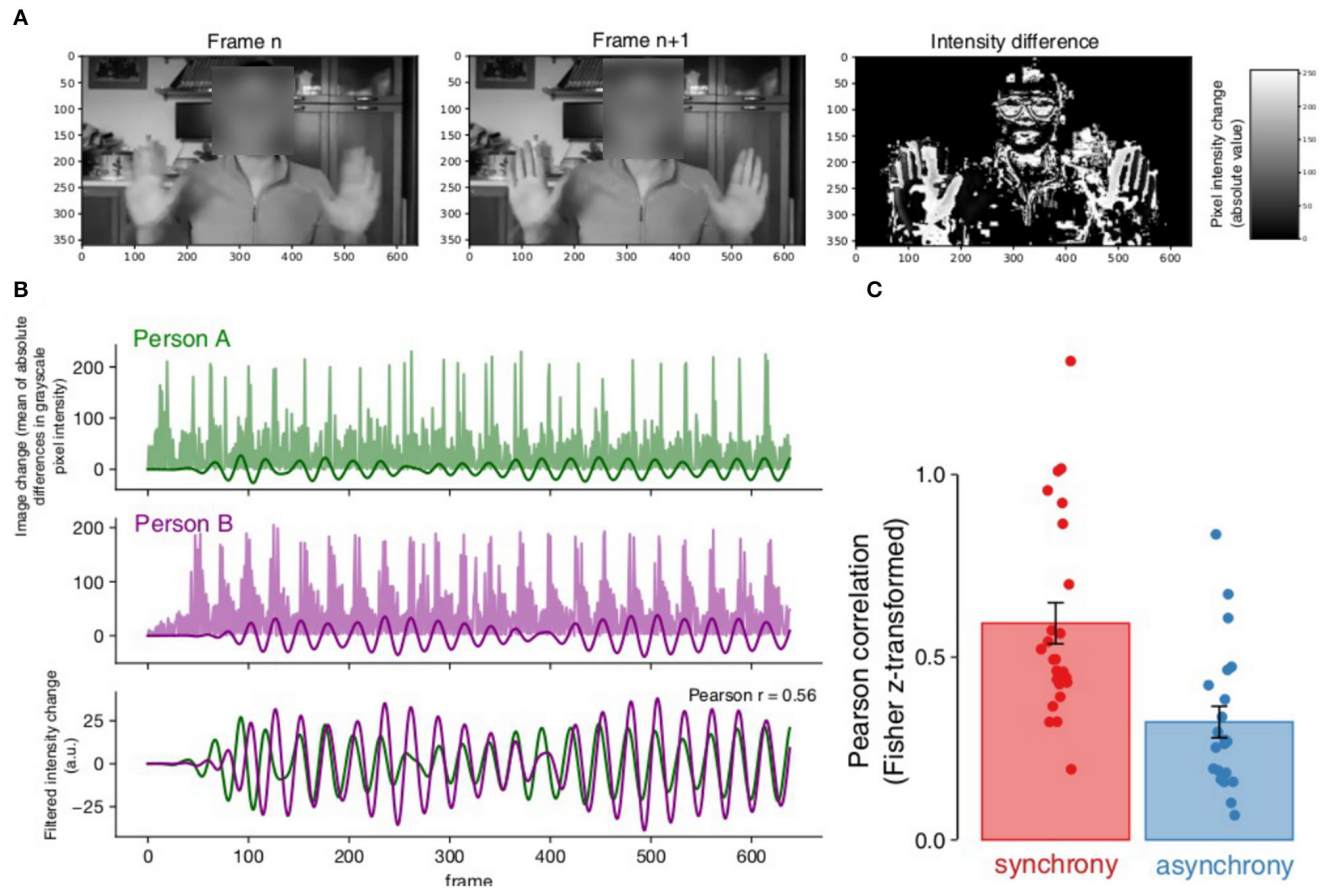
Manipulation check. Manipulation check indicating that the nominal synchrony dyads moved more in synchrony than the asynchrony condition. See text for detail. **(A)** Differences in pixel intensity were calculated across the image and averaged. **(B)** Raw mean absolute image change showed periodic patterns in both participants (bar graph in the background) that were extracted using bandpass filtering (line in the foreground). Pearson correlation was calculated between these traces (bottom panel). **(C)** The dyads in the nominal synchrony condition showed greater correlation among their movement traces than participants in the asynchrony condition. Bars represent average, error bars indicate standard error of the mean and dots indicate individual dyads.

In order to assess the amount of synchrony in the experimental groups (nominal synchrony and asynchrony conditions) we performed an analysis that was inspired by Motion Energy Analysis (Ramseyer, 2020). For each dyad, we extracted the part of the video recording where participants made movements paced by the metronome. We analyzed each of the two persons' video separately, converted into grayscale (Figure 3A). For each consecutive two frames, we calculated the pixel-by-pixel intensity difference. Taking the mean absolute value of these differences across the frame, this yielded a single value estimating the overall amount of change in the frame relative to the next. The corresponding time course was filtered (5th order Butterworth bandpass filter 0.75–1.25 Hz) to extract the periodicity in the vicinity of the pacing frequency (Figure 3B). In order to account for possible small delays in the signal, we calculated the maximum Pearson cross-correlation between the signals with a maximum shift of 25 frames (1 s). This yielded a single Pearson correlation value per dyad which was Fisher r-to-z transformed and compared between synchrony and asynchrony conditions (Figure 3C). The image change time courses showed greater correlation in the synchrony condition (Pearson *z* mean = 0.59, *SD* = 0.28) than in the asynchrony condition (Pearson *z* mean = 0.32, *SD* = 0.20) [*t* (41.50) = 3.81, *p* < 0.001, Cohen *d* = 1.11, 95% CI (0.47, 1.76)]. This suggests that the nominal synchrony dyads indeed moved more synchronously than dyads in the nominal asynchrony condition.

### Synchrony and Social Affiliation

**Figure 4:**
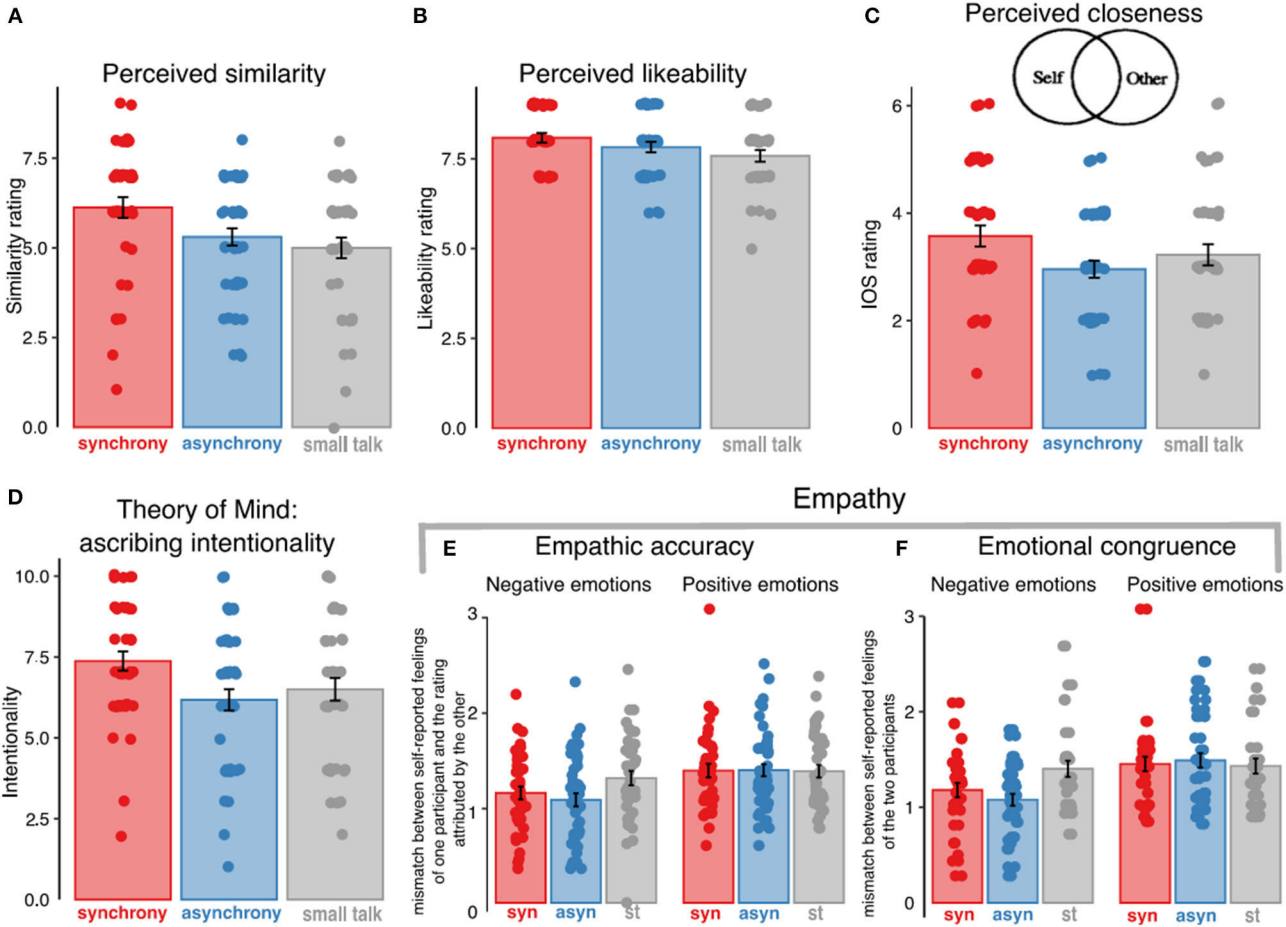
Effects of synchrony on social affiliation, empathy and theory of mind. Participants in the synchrony group reported greater similarity **(A)**, likeability **(B)**, and closeness **(C)** to their partners than participants in asynchrony and small talk groups. Closeness was measured using the Inclusion of Other in the Self (IOS) scale of which a sample item is shown in the inlay. Participants in the synchrony group attributed greater intentionality to triangles in a video **(D)**. For the empathic measures, empathic accuracy **(E)** and emotional congruence **(F)** results are shown separately for negative and positive emotions, and no group differences were found. In all panels dots represent participants' single scores; bars represent group averages and error bars the standard error of the mean. syn, synchrony; asyn, asynchrony; st, small talk.

We found a significant effect of the experimental group on perceived similarity, *F* (2, 122) = 4.14, *p* = 0.014, $\eta_{p}^{2}$ = 0.68 (Figure 4A). Planned contrasts revealed statistical trends toward significance with participants in the synchrony condition rating their partners as more similar to themselves than participants in the asynchrony condition, *t* (122) = 2.17, *p* = 0.063, and participants taking part in the synchrony and asynchrony groups with respect to those belonging to the small talk one, *t* (122) = 2.13, *p* = 0.067. The ANOVA on likeability scores showed a statistical trend toward significance for the effect of the experimental group, *F* (2,110) = 2.82, *p* = 0.063, $\eta_{p}^{2}$ = 0.45 (Figure 4B). Planned contrasts showed no significant difference between synchrony and asynchrony groups *t* (110) = 1.18, *p* = 0.42. The synchrony and asynchrony vs. small talk contrast was marginally significant, with synchrony/asynchrony participants condition showing higher scores than those in the small talk condition, *t* (110) = 2.16, *p* = 0.64. For perceived closeness, results revealed a significant main effect of experimental group *F* (2, 122) = 3.07, *p* = 0.050, $\eta_{p}^{2}$ = 0.05 (Figure 4C). Planned contrasts showed that the synchrony group reported more closeness than the asynchrony group, *t* (122) = 2.48, *p* = 0.029. The contrast between synchrony/asynchrony and small talk groups was not significant, *t* (117) = 0.80, *p* = 0.67. No significant difference between groups was found on the future friendship question, *F* (2, 121) = 1.66, *p* = 0.20, $\eta_{p}^{2}$ = 0.03.

Overall, these data showed that participants in the synchrony condition were more likely to perceive their experimental partner as close and, marginally, as more similar than in the asynchrony group but did not rate more liking or future friendship possibility.

### Synchrony and Empathic Accuracy, Emotional Congruence

For empathic accuracy, we first conducted a 3 x 2 mixed ANCOVA with group as between-subject variable, valence of the emotions (positive and negative) as within-subjects factor and empathic accuracy as dependent variable (controlling for gender). Results showed no significant effect of the experimental group, *F* (2, 120) = 1.30, *p* = 0.275, $\eta_{p}^{2}$ = 0.02, and no significant interaction, *F* (2, 120) = 1.75, *p* = 0.178, $\eta_{p}^{2}$ = 0.03 (Figure 4E). A significant effect was found for the valence of the emotions, *F* (1, 120) = 19.75, *p* < 0.001, $\eta_{p}^{2}$ = 0.14, indicating that empathic accuracy was greater for negative than for positive emotions. Since the main effect of group was not significant, we proceeded to calculate the Bayes Factor of the principal contrast of interest between synchrony and asynchrony and found BF_10_ = 0.23 for positive and BF_10_ = 0.29 for negative emotions. This indicates moderate evidence in favor of the null hypothesis that there is no difference between synchrony and asynchrony groups in empathic accuracy for both positive and negative emotions.

For emotional congruence there was no significant effect of group, *F* (2, 118) = 1.76, *p* = 0.176, $\eta_{p}^{2}$ = 0.03, but a significant main effect of the valence of the emotions, *F* (1, 118) = 27.68, *p* < 0.001, $\eta_{p}^{2}$ = 0.19, with emotional congruence being greater for negative emotions than for positive emotions (Figure 4F). The interaction between experimental group and valence of emotions was significant, *F* (2, 118) = 3.71, *p* = 0.027, $\eta_{p}^{2}$ = 0.06. We then followed up with two separate ANCOVAs on positive and negative emotional congruence, respectively. For positive emotional congruence, the effect of group was not significant, *F* (2, 118) = 0.23, *p* = 0.79, $\eta_{p}^{2}$ = 3.92, but for negative emotional congruence it was, *F* (2, 118) = 5.53, *p* = 0.005, $\eta_{p}^{2}$ = 0.09. Planned group contrasts for negative emotional congruence revealed no significant difference between synchrony vs. asynchrony group, *t* (118) = 1.05, *p* = 0.50, but a significantly greater emotional congruence in the synchrony and asynchrony vs. the small talk group, *t* (118) = −3.11, *p* = 0.005. In sum, we did not find differences in emotional congruence between synchrony and asynchrony groups, whereas negative emotional congruence was greater in synchrony/asynchrony groups relative to the small talk. To assess the evidence for the finding that emotional congruence does not differ between groups, we calculated the Bayes Factor of the difference between synchrony and asynchrony groups and found BF_10_ = 0.24 for positive (moderate evidence) and BF_10_ = 0.37 (some evidence) for negative emotions. Overall, synchrony and asynchrony groups did not differ in assessing or sharing others' emotions.

### Synchrony and Theory of Mind

The ANCOVA (controlling for gender) on the Triangle intentionality attribution scores showed a significant main effect of experimental group, *F* (2, 122) = 3.39, *p* = 0.028, $\eta_{p}^{2}$ = 0.06 (Figure 4D). Planned contrasts revealed that participants in the synchrony condition were more likely to attribute intentions to the triangles than participants in the asynchrony condition, *t* (122) = 2.65, *p* = 0.009. No significant difference was found between the synchrony and asynchrony vs. the small talk group, *t* (122) = 0.71, *p* = 0.73. For the Mental State Attribution questionnaire, no significant differences between groups were found, *F* (2, 120) = 1.49, *p* = 0.23, $\eta_{p}^{2}$ = 0.02. Overall, these results showed that participants in the synchrony group showed greater tendency to describe the video of the triangles in mental state terms than in the asynchrony group, but we did not find evidence that they were more likely to see other people as carrying mental states.

## Discussion

The present study investigated the effect of synchronous movement on social affiliation, empathy and ToM. We tested unacquainted participants who met on-line in dyads and performed 3 min of synchronous or asynchronous movements, or a semi-structured small talk conversation. We examined self-reported social affiliation with the dyadic partner (similarity, closeness, likeability, possibility of a future friendship), ToM (attribution of intentionality and mental states, using an objective and a subjective measure) and objective measures of empathy (empathic accuracy and emotional congruence). First, we found that participants in the synchrony group reported greater closeness to their partner relative to participants in the asynchrony and small talk groups, and a statistical trend toward greater similarity. Second, participants in the synchrony group were more likely to attribute intentions to abstract moving shapes than participants in the asynchrony and small talk groups. No differences were found between groups in attributing mental states to their partners. Finally, participants in the synchrony and asynchrony groups showed similar levels of accuracy in estimating (empathic accuracy) and sharing (emotional congruence) of their partner's emotions.

### Synchrony and Social Affiliation

The present study found that the synchrony group reported greater closeness toward their partner relative to the asynchrony group, in line with prior in-person studies, whereas for a number of other social affiliation measures (similarity, likeability and future friendship) results were only marginal or absent. This suggests that in online encounters at least a subset of in-person effects of synchrony on social affiliation can be replicated.

Our finding that synchrony increases perceived closeness fits with existing studies that have typically been conducted in person (Paladino et al., 2010; Vacharkulksemsuk and Fredrickson, 2012; Reddish et al., 2013; Fessler and Holbrook, 2014; Lumsden et al., 2014; Dong et al., 2015; Tarr et al., 2016). Taken together these results show that moving in synchrony makes people feel closer to each other and this is true both when participants met online and offline. The effect of synchrony on closeness is in accordance with the self-other blurring hypothesis. According with this view, synchronizing with someone, compared to other coordinated behavior such as asynchronous or sequential action, blurs the boundaries between the self and other (Hove, 2008). This is thought to be because typically during synchronous movement, two people perform the same movement at the same time. This match between one's own action and the observation of another performing the same action makes it more difficult for the brain to distinguish between self and other (Paladino et al., 2010; Mazzurega et al., 2011; Tarr et al., 2014; Rennung and Göritz, 2016). This blurring of the self-other boundary would lead to greater perceived closeness or even a sense of oneness with a group (Swann et al., 2012). As for similarity, our results are partially in line with previous literature which reported an increase of the sense of similarity after synchronizing with someone compared to asynchronous or control conditions (Wiltermuth and Heath, 2009; Schachner and Garvin, 2010; Valdesolo et al., 2010; Valdesolo and DeSteno, 2011; Dong et al., 2015). The present study also indicated a trend for greater similarity in the synchrony group (relative to the asynchrony group) although this did not reach statistical significance. With regard to likeability, our data did not replicate a prior finding of an increase in liking after synchronous movement (Valdesolo and DeSteno, 2011).

We did not find an effect of synchrony on the perceived possibility to develop a future friendship with the experimental partner. As far as we know, our study is the first one testing whether this indicator of social affiliation is affected by synchrony. We expected participants in the synchrony group to be more willing to become friends than participants in the asynchrony group. This is because synchrony typically increases a range of social affiliation dimensions (Hove and Risen, 2009; Paladino et al., 2010; Valdesolo et al., 2010; Vacharkulksemsuk and Fredrickson, 2012; Reddish et al., 2013; Tarr et al., 2015, 2016) that presumably make one more likely to want to form a future friendship (Sedikides et al., 1999). However, we were not able to find support for this hypothesis.

This pattern of findings may reflect one of two hypothetical scenarios. A first possibility is that in-person, synchrony can affect a range of social affiliation measures, whereas online it affects only closeness. We speculate that the reason other affiliation measures (similarity, likeability) may not be affected by online synchrony in the same way as in-person synchrony is because of a lack of relevant visual cues, such as eye contact and physical proximity. Indeed, previous literature suggested that during in-person interactions, similarity and likeability are sensitive to such information (Peters, 2007). However, it remains challenging for this account to explain why the effect of online synchrony would be specific to closeness and not hold for related measures such as similarity and likeability. A second hypothetical scenario is that online synchrony actuality affects all measures of social affiliation, but the effects on likeability and the possibility of a future friendship were smaller and, therefore, did not reach significance here. This account is in line with theories of synchrony's social effects in which synchrony should affects multiple measures of social affiliation across the board (Hu et al., 2022). It remains unclear whether the effect on likeability and the possibility of a future friendship would be smaller. Since prior literature has typically merged all these variables (similarity, closeness, etc.) into a single, aggregate social affiliation measure, we do not have prior estimates for the effect sizes on the individual variables.

Our study is the first, to our knowledge, to document the social effects of synchrony in people meeting through video conferencing. There are some distantly related studies that found that synchronous movements between avatars (stick figures) can lead to greater perceived closeness and sympathy ratings (Stupacher et al., 2017a,b, 2020, 2021). If indeed such results rely on similar synchrony-to-social processes as operating between humans, as is sometimes tacitly assumed, then this lends credence to the idea that social effects of synchrony are not restricted to humans meeting in person. However, these studies differ in important ways from the present work, that makes such extrapolations tentative.

To summarize, our findings suggest that synchrony increases closeness in online environments. On the contrary, findings of other social affiliation measures were less clear: no detected difference in likeability and future friendship and only a statistical trend on similarity.

### Synchrony and Empathy

Our data showed no difference between synchrony and asynchrony conditions for both empathic accuracy - the accuracy in understanding other's emotions - and emotional congruence - sharing emotions with another person.

It has been proposed that moving in synchrony would be associated with greater emotion sharing (Shamay-Tsoory et al., 2019). Indeed, on a subjective level this has been confirmed by a study in which participants taking part in a unilateral synchronization estimated that they shared emotions with their partners more than participants in an asynchrony condition (Koehne et al., 2016), although (Baimel et al., 2018), asking a similar question, did not find this effect. Here we tested whether this effect may hold on an objective level as well, by directly comparing the ratings of felt emotions by the two partners (emotional congruence). We found no difference between the synchrony and asynchrony conditions. Taken together, this suggests that although participants may have thought they shared the emotions of their partner (based on prior work), they did not actually do so (based on the present study). Synchrony may, thus, introduce a positive subjective bias in emotional congruence, making us believe we share more emotions with others than we really do. We speculate that this positive subjective bias may help us feel closer to the people we synchronize with, and that it can perform this function without requiring that we actually feel the same. Since in this study we did not collect subjective measures of emotion sharing we cannot test this association between closeness and subjective emotion sharing directly. The mismatch between subjective and objective measures of emotion sharing is reminiscent of similar mismatches that have been documented in the empathic accuracy literature. Indeed, subjective measures of empathic accuracy (thinking we know what the other feels) often differ from objective measures of empathy (actually knowing what the other feels) (Zaki et al., 2008; Murphy and Lilienfeld, 2019). If indeed synchrony affects subjective but not objective emotion sharing, future theoretical accounts of synchrony may need to draw this distinction and explain why that is the case.

In terms of accuracy of understanding others' emotions (empathic accuracy), prior studies like us did not find an effect of synchrony. However, empathic accuracy was typically measured using tasks that evaluate the extent to which participants correctly identified the emotions of people other than the synchronizing partners, such as for example when inferring emotions from a picture of somebody's eyes (Baimel et al., 2018). This left open the possibility that synchrony selectively boosts empathy toward the people we synchronize with (as has similarly been demonstrated in the case of helping behaviors, Cirelli et al., 2014; Cirelli, 2018), which is what we tested here. However, empathic accuracy toward the synchronization partner was not significantly different between the synchrony and asynchrony conditions. One other study focusing on spontaneous not imposed synchronization, investigated empathic accuracy toward the synchronization partner (Fujiwara and Daibo, 2021) and similarly found no relation to spontaneous synchronization. This study used a composite measure of empathic accuracy that included awareness of both thoughts and emotions. As a result, it was not clear whether synchrony might have specifically affected empathy for emotions. Our result provides evidence that this is not the case. Taken together, converging lines of evidence including our study reveal that synchrony has little if any effect on empathy, regardless of how synchrony was achieved (spontaneous vs. imposed) or toward whom empathy was directed (synchrony partner or others).

The lack of effect of synchrony on empathic accuracy in our study may help nuance more broad-stroke claims that effects of prolonged music training on objective measures of empathy are mediated by synchrony. Long-term music training has been associated with some effects on empathic functioning (Thompson et al., 2003; Rabinowitch et al., 2013) and some works have speculated this is driven by synchrony inherent in the musical training (Rabinowitch, 2017). However, other components of long-term music training are likely to be responsible for these boosts in empathy. One component may be musical material itself. Indeed, studies on social affiliation show that presenting music, as opposed to a simple metronome or silence, leads to greater closeness and likeability, and that this is true regardless of synchrony (Stupacher et al., 2017a). Perhaps a similar knock-on effect would hold for empathy, namely that regardless of synchrony, music would boost empathic accuracy. This could explain why music training could promote empathic accuracy when synchrony alone does not. Another component is the long-term nature of the music training tested in the aforementioned studies. Indeed, improving empathic accuracy by means other than synchrony or music, usually takes extended periods of time (Mascaro et al., 2013; Lobchuk et al., 2016; Kraus, 2017; Haut et al., 2019) except for very short pharmacological interventions (Bartz et al., 2010). Thus, it is possible that our synchrony intervention was too short to have a reliable impact on empathic accuracy.

Overall, our study along with previous research, paints a picture where synchrony is not linked with greater accuracy in inferring or sharing others' emotions.

### Synchrony and Theory of Mind

Synchronous participants attributed more to mental states to abstract geometrical shapes but not to their partner. Attribution of mental states to the partner was measured using a questionnaire. Attribution of mental states more broadly was tested using a task involving a set of videos of geometrical shapes moving so as to suggest human interaction, in the absence of any contextual verbal or non-verbal cues other than movement. We screened participants' descriptions of these videos for the degree of intentionality they attributed to the shapes and found that this was higher for participants in the synchrony compared to the asynchrony group. This suggests that synchrony has an effect on the attribution of intentionality. This result at first glance may seem to contradict a prior study that found that the quality of synchrony did not correlate with ToM performance (Koehne et al., 2016). In that study, participants, after a unilateral synchronization task, were shown videos of four human characters getting together for a dinner party and then had to respond to questions as to what the different characters were thinking, feeling or intending. The individual differences in scores on this task (Movie for Assessment of Social Cognition, MASC; Dziobek et al., 2006) did not correlate with the accuracy of the synchronization. We argue that this finding does not contradict but complements the present results. The Koehne study assessed the accuracy with which participants can infer other people's mental states which is a different aspect of ToM from what we tested here, namely the extent to which participants spontaneously ascribe mental states independently of whether they do so accurately or not. Thus, a preliminary picture seems to emerge from studies so far on synchrony and ToM and empathy where some subjective measures are affected by synchrony, but no accuracy measures are: no effect on empathic accuracy (our study and Fujiwara and Daibo, 2021), nor emotion sharing (our study), nor reading-the-mind-in-the-eyes (Baimel et al., 2018) nor assessing intentions in a movie (MASC; Koehne et al., 2016).

The Triangle task has been successfully used in studies involving typical adults (Devine and Hughes, 2019; Ceccato et al., 2020) and has shown convergent validity with other ToM tasks (Devine and Hughes, 2019; Lecce et al., 2021). Previous research has suggested that the detection of agency on the basis of motion cues, such as velocity changes and movements that appear interactive, may be a precursor of ToM (Blakemore et al., 2003) and relies on brain areas involved in understanding social information in human motion (Castelli et al., 2002). Given that previous research has shown that performance in the Triangle task is associated with social competence (Ceccato et al., 2020), at least in children, possibly the differences we found in this task in adults could also be relevant for social interactions.

Our results suggest that moving in synchrony has effects on other tasks that no longer involve the person we synchronized with (in our case, the Triangle task). This is analogous to prior work documenting effects of synchrony on prosocial behavior, showing that these effects extend beyond the particular people we synchronize with (Reddish et al., 2013; but see also Cirelli et al., 2014; Cirelli, 2018). However, in the present study, we did not observe an effect of synchrony on mental state attribution for the person synchronized with. This contrasts with results of Baimel et al. (2018) who reported that participants in the synchrony condition were more likely to attribute mental states to their partners than participants in the asynchrony condition. This failure to replicate the prior result could be, we speculate, because the effect of synchrony on ToM is smaller in dyads (as we tested) than in triads (as in Baimel et al., 2018). Indeed, although not addressing mental state attribution, previous literature has indicated that group size may moderate synchrony effects on other measures of social affiliation (prosocial behavior and positive affect): the larger the group, the bigger the effect (Mogan et al., 2017). Extrapolating from these findings, we speculate that in our case, the group size (dyads) may have been too small to yield effects that were observed previously for triads.

Our study taken together with prior work suggests that the link between synchrony and ToM may go both ways. We tested the effect of synchrony on ToM. Prior work has documented the opposite direction: whether pre-existing ToM differences lead to greater synchrony. Novembre et al. (2019) divided participants into high and low empathic perspective taking pairs (based on scores of the *perspective taking* subscale of the Interpersonal Reactivity Index, IRI). Participants in the high empathy group synchronized more accurately in time than participants with low empathy. In another set of studies, Autism Spectrum Disorder (ASD) individuals had lower synchronization performance relative to neurotypical controls (Gowen and Miall, 2007; Fitzpatrick et al., 2013, 2017; Marsh et al., 2013). One possibility is that this synchronization difference is related to the documented difference in mentalizing abilities in ASD individuals. However, other differences between typical and ASD individuals other than mentalizing could explain this effect, such as social perceptual processes (Klin et al., 2002). Taken together, existing literature may suggest a bidirectional relationship between synchrony and ToM, as has been proposed before (Shamay-Tsoory et al., 2019). However, the studies for the two directions of this relationship did not use the same measures of ToM (subjective vs. performance-based) and synchrony (spontaneous vs. imposed). This leaves open the possibility that each direction of the relationship holds only for a specific ToM component considered or on the way that synchrony was manipulated, and thus it would not be fully bidirectional. It would be interesting for future studies to further hash out this relationship between ToM and synchrony by evaluating whether an intervention that increases ToM leads to greater synchrony after relative to the before.

In sum, our findings suggest that synchrony affects the tendency to spontaneously ascribe intentionality to moving geometric shapes but not the self-reported attribution of mental states to the synchronizing partner.

### Secondary Findings

Participants in the synchrony and asynchrony conditions together, relative to the small talk condition, showed greater emotional congruence (emotion sharing) for negative emotions. This finding was selective: no differences were found between the synchrony and asynchrony groups, nor differences in empathic accuracy, nor differences for positive emotional congruence. The small talk condition was included as a benchmark of the social effects of a relatively natural, normal interaction. The emotional congruence effect observed can be due to many factors and our data do not allow us to determine which. For instance, the synchrony and asynchrony conditions both involved prescribed periodic movements paced by auditory sounds whereas the small talk condition did not. The small talk condition involved verbal interaction whereas the other conditions did not. Or again, the difference in emotion sharing could be due to the fact that the synchrony and asynchrony conditions both performed the same movement, thus giving rise to a mirroring of sort (even though not necessarily at the same time). The small talk condition allowed for sharing of personal details whereas the synchrony/asynchrony conditions did not. Note also that the small talk conversation could have given rise spontaneously to more or less synchronous movements, as has been previously observed, due to a number of factors (Fusaroli et al., 2013; Varlet et al., 2014; Schmidt and Fitzpatrick, 2019; Fujiwara and Daibo, 2021). Any of these differences, or yet others we have not listed, could be responsible for the emotion sharing difference observed here.

Both empathic accuracy and emotional congruence were greater for negative than for positive emotions. This was true across groups and, thus, not affected by synchrony or small talk. We are not aware of studies systematically investigating whether positive or negative emotions are more prone to be shared. Perhaps what we observed is because negative emotional information tends be more salient (Baumeister et al., 2001; Vaish et al., 2008; Fessler et al., 2015), since individuals in many cases tend to prefer sharing positive emotional content (Gillath et al., 2005). Prior literature has drawn distinctions between empathy for negative and positive emotions (Andreychik and Lewis, 2017). Empathy for others' positive emotions is associated with dispositional positive emotionality (propensity to experience frequent, intense, or enduring positive affect), engagement in behaviors aimed to enhance others' positive emotions and with engaging in random acts of kindness (Andreychik and Migliaccio, 2015). Empathy for others' negative emotions is associated with helping others when it is framed as avoiding them to suffer (Andreychik and Lewis, 2017). Our data does not allow us to distinguish whether greater sharing of negative emotions is a general phenomenon or an artifact of the conversation that we had our participants engage in. In our study, participants always first talked about a negative event, which may have led to a greater overall negative tone to the conversation which was reflected in greater sharing of negative emotions.

### Future Directions and Limitations

Synchrony can occur spontaneously or intentionally. When intentional, it may come about through different processes that have different social outcomes. Cacioppo et al. (2014) distinguish between orchestration (when multiple participants follow a common stimulus), unilateral entrainment (when one of the participants is a leader that the others follow) or reciprocal entrainment (when the participants mutually adjust their movements). Although a growing literature documents differences in dynamics between these kinds of synchrony, the social effects of each have received little systematic comparison. The present study focuses on synchronizing with an imposed metronome (i.e., orchestration) as a number of prior studies did (Macrae et al., 2008; Hove and Risen, 2009; Valdesolo and DeSteno, 2011; Reddish et al., 2013, 2016; Baimel et al., 2018). Although this allowed us greater control in terms of tempo over time and across dyads, it leaves open the possibility that social effects would have been different had we studied spontaneous or bilateral entrainment. Future studies could explore these possibilities. At least in principle, it is possible that bidirectional synchrony would have a stronger effect on ToM and empathy than a synchrony condition in which participants are required to synchronize with an external metronome because in that case there is no shared goal. A prior study that focused on social effects other than ToM and empathy indeed found that bidirectional synchronization (shared goal) has larger effects on measures of trust, cooperation and perceived similarity and closeness toward the synchronizing partners than a unilateral synchrony with a metronome (Reddish et al., 2013).

The present study did not include pre-manipulation measures of the variables of interest so we could not control for potential differences in baseline levels of social affiliation, ToM and empathy. Second, we did not measure the fine-grained accuracy of synchronous movements in each pair of participants (e.g., the time differences between the movements of the two partners). This could be helpful in future studies to ask whether pairs who are more accurately synchronous in time also yield greater effects on social affiliation and social cognition. Third, possibly our sample size was too small to detect more subtle effects. Further, the absence of effects on empathic measures (empathic accuracy and emotional congruence) reported here could be due to the online nature of the test. It is possible, at least in principle, that these effects would be observed had participants met in person. We think this possibility is less likely, given the degree of convergence between our result and that of prior studies on other social measures. However, future studies could test this more directly.

Our study was conducted during a time of exceptional social circumstances. In the spring of 2020, as data was collected, our Italian participants were in a state of lockdown where in-person social interactions were in many cases severely limited. In that situation online interactions may have provided the social support that is otherwise obtained in-person meetings (Pancani et al., 2021; Marinucci et al., 2022). This could have biased the results of the present study: perhaps participants were more open to engage in online interpersonal interactions than they would have been under conditions of less social isolation. Note that boosting closeness under these exceptional circumstances also highlights the potential of our intervention: that online encounters can, through even brief periods of synchronous movement, become a source of interpersonal closeness. Closeness, in turn, has been associated with greater emotional wellbeing during the pandemic (Cavallini et al., 2021). In this way, online social interactions may be beneficial when in-person interactions are not available (Waytz and Gray, 2018).

In conclusion, our study found that synchronous movements during online encounters increased a number of measures of social affiliation and Theory of Mind, but not empathic accuracy or emotion sharing. These results highlight the potential of synchronous movement in online encounters to affect a subset of social cognition and affiliation measures.

## Data Availability Statement

The datasets supporting the conclusions of this article are publicly available online via https://osf.io/Jchzb/.

## Ethics Statement

The studies involving human participants were reviewed and approved by Ethics Committee of the Department of Brain and Behavioral Sciences of the University of Pavia. The patients/participants provided their written informed consent to participate in this study.

## Author Contributions

Conceptualization and writing: CB, SL, and FV. Investigation and data collection: CB. Supervision: SL and FV. All authors contributed to the article and approved the submitted version.

## Conflict of Interest

The authors declare that the research was conducted in the absence of any commercial or financial relationships that could be construed as a potential conflict of interest.

## Publisher's Note

All claims expressed in this article are solely those of the authors and do not necessarily represent those of their affiliated organizations, or those of the publisher, the editors and the reviewers. Any product that may be evaluated in this article, or claim that may be made by its manufacturer, is not guaranteed or endorsed by the publisher.
