# Supplementary material for: Synchrony During Online Encounters Affects Social Affiliation and Theory of Mind but Not Empathy
Source: Front Psychol. 2022 Jul 11;13:886639. doi: 10.3389/fpsyg.2022.886639 (PMC9450704; doi:10.3389/fpsyg.2022.886639)
Supplement: Supplementary file 1 [file Data_Sheet_1.PDF]

Supplementary materials

The main analyses of this manuscript used a priori planned contrasts to first compare synchrony vs. asynchrony conditions and secondly compare the synchrony and asynchrony conditions together with the small talk condition. For reference we include in here the synchrony vs. small talk and asynchrony vs. small talk contrasts. In the table below the follow-up analyses are included for variables where AN(C)OVA was significant (Supplementary Table 1).

Supplementary Table 1. *Comparison between planned orthogonal contrasts and the post-hoc comparison of the three experimental conditions.*

variable	**Planned orthogonal contrasts**		**Post-hoc comparison of all three conditions**		
	Synchrony vs. asynchrony	Synchrony vs. asynchrony	Synchrony vs. small talk	Asynchrony vs. small talk	[Synchrony & Asynchrony] vs. small talk
Closeness	$t(122) = 2.48$, $p = .029$	$t(122) = 2.48$, $p = .043$	$t(122) = 1.34$, $p = .453$	$t(122) = -1.09$, $p = .0625$	$t(122) = 0.17$, $p = .982$
Similarity	$t(122) = 2.17$, $p = .063$	$t(122) = 2.17$, $p = .092$	$t(122) = 2.87$, $p = .014$	$t(122) = 0.8$, $p = .81$	$t(122) = 2.13$, $p = .070$
Likeability	$t(108) = 1.24$, $p = .391$	$t(108) = 1.24$, $p = .525$	$t(108) = 2.32$, $p = .065$	$t(108) = 1.15$, $p = .584$	$t(108) = 2.01$, $p = .093$
ToM Intentionality	$t(122) = 2.65$, $p = .019$	$t(122) = 2.65$, $p = .027$	$t(122) = 1.88$, $p = .174$	$t(122) = -0.70$, $p = .864$	$t(122) = 0.71$, $p = .732$
Emotional congruence (negative emotions)	$t(118) = 1.05$, $p = .503$	$t(118) = 1.05$, $p = .650$	$t(118) = -2.20$, $p = .087$	$t(118) = -3.29$, $p = .004$	$t(118) = -3.11$, $p = .005$

Note that the “synchrony vs. asynchrony" contrast appears in both analysis strategies (post-hoc vs. planned orthogonal contrasts). The p-values in this case differ because multiple comparisons corrections apply over different numbers of contrasts: 2 vs. 3.
